# Wakayama symposium: role of canonical Notch signaling in conjucntival goblet cell differentiation and dry eye syndrome

**DOI:** 10.1186/s12886-015-0136-6

**Published:** 2015-12-17

**Authors:** Chia-Yang Liu

**Affiliations:** Edith J. Crawley Vision Research Center/Department of Ophthalmology, College of Medicine, University of Cincinnati, Cincinnati, OH 45267-0838 USA; Present Address: Opt511, Indiana University School of Optometry, 800 E. Atwater Avenue, Bloomington, IN USA

**Keywords:** Dry eye, Goblet cells, Ocular surface, Morphogenesis, Notch signaling

## Abstract

This review summarizes a recent finding regarding the intrinsic canonical Notch signaling pathway in regulating normal ocular surface morphogenesis and its role in the pathogenesis of goblet cell deficiency-associated keratoconjunctivitis sicca (KCS, or dry eye). Specifically, we used novel transgenic mice to investigate the mechanism of how the Notch1 activation may serve as the upstream control of expression of transcription factors Krüppel-like factors 4 or 5 (Klf4 or Klf5) which in turn controls goblet cell differentiation and activates mucin 5/ac synthesis during ocular surface morphogenesis.

## Introduction

Ocular surface goblet cells are polarized epithelial cells scattered along the conjunctival epithelium in which their numbers in bulbar (or ocular) conjunctiva at the fornix area are much more abundant than those in palpebral (or tarsal) conjunctiva. Conjunctival goblet cell is the major cell type that synthesizes and secrets mucins for the maintenance of ocular surface integrity. Lack of mucins in tear film due to goblet cells abnormality causes dry eye syndrome (DES) and affects millions of people’s vision and life [1–3]. The lack of knowledge regarding the regulatory mechanisms by which conjunctival epithelial cells differentiate to form goblet cells hampers the development of treatment regimens for DES.

The cellular mechanism underlying goblet cell formation and related pathogenesis in the ocular surface epithelia is poorly understood. A recent report showed that members of the canonical Notch signaling pathway i.e., Notch1, Notch2, Notch3, Jagged1, Dll1, were significantly down-regulated in dry eye as compared to the non-dry eye conjunctival epithelia [4]. This result suggested that Notch deregulation played an important role in the pathogenesis of human dry eye syndrome. Nevertheless, the specific function of canonical Notch signaling pathway in ocular surface goblet cell fate determination has not been well documented or explored in vivo. Therefore, it is important to understand the molecular basis of how epithelial phenotypes of nonkeratinization and mucin expression are modulated by Notch signaling to endow the mucous ocular surface with sufficient moisture that is essential to maintain the integrity and health of the corneal and conjunctival surface.

### Canonical Notch signaling

Notch signaling is a central mediator of short-range inter-cellular communication in metazoans [5, 6]. It regulates cell fate decision and plays critical roles in controlling goblet cell differentiation in the gut epithelium [7]. Moreover, Notch activation has been postulated to contribute in the maintenance of corneal [8–11] and conjunctival [12, 13] phenotypes. The Notch receptor exists at the cell surface as a proteolytically cleaved heterodimer consisting of a large ectodomain and a membrane-tethered intracellular domain. Ligands of the Delta-like (DLL1, DLL3, DLL4) and Jagged (JAG1 and JAG2) families interact with receptors of Notch family (NOTCH1-4) on an adjacent cell. The ligand-receptor interaction induces further proteolytic cleavages of the Notch that release the Notch intracellular domain (NICD) from the cell membrane. The NICD translocates into the nucleus, where it forms a complex with the recombination signal binding protein for immunoglobulin kappa J region (RBP-Jκ) protein, displacing a histone deacetylase (HDAc)-co-repressor (CoR) complex from the RBP-Jκ protein. Components of an activation complex, i.e., transcriptional co-activators mastermind-like protein 1 (MAML1) and histone acetyltransferase (HAT) p300, are recruited to the NICD-RBP-Jκ complex, leading to the transcriptional activation of Notch target genes such as Hes1 (hairy and enchancer of split-1)/Hey1 (Hairy/enhancer-of-split related with YRPW motif 1) and others [14].

### Findings in transgenic mouse model

To investigate Notch function in ocular surface morphogenesis, we generated a doxycycline (Dox)-inducible compound transgenic mouse strain, designated as K14-rt/TC/Rosa^LSL-dnMAML1^ which harbored three following transgenes:

1. *K14-rtTA* (*K14-rt*): Keratin 14-promoter-driven reverse tetracycline trans-activator [15],
2. *tet-O-Cre* (*TC*): Cre recombinase under the control of a tetracycline-responsive promoter element (*TRE; tet-O*) [16], and
3. *Rosa*^*LSL-dnMAML1*^: a fusion cDNA cassette LSL-dnMAML1 consisting of two LoxP (L) sites flanked stop (S) sequence followed by a dominant negative MAML1 under the control of the mouse *Rosa 26* gene locus [17].

**Fig. 1:**
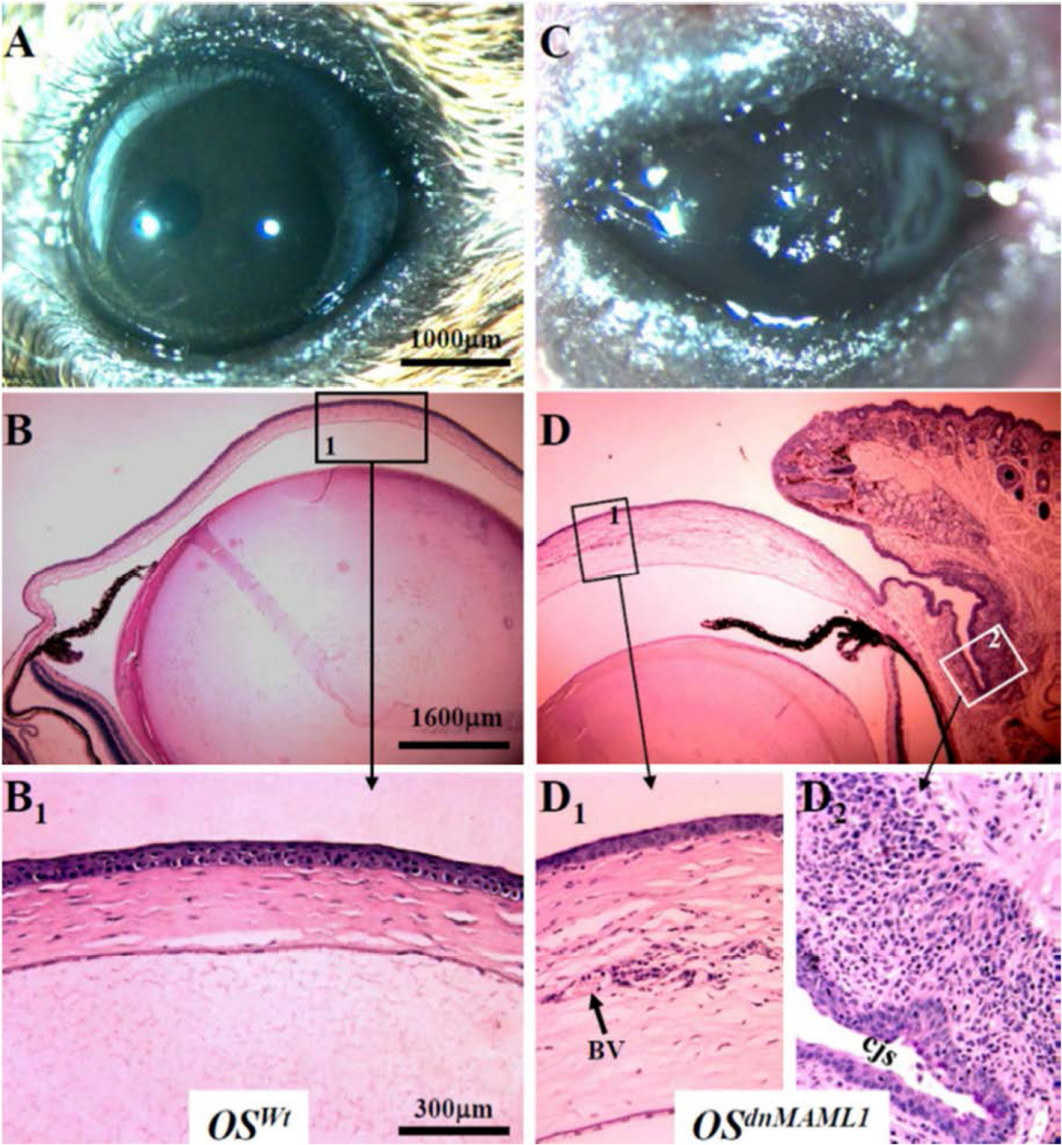
Expression of dnMAML1 led to irreversible loss of goblet cells. TC/RosaLSL-dnMAML1 (OS^wt^) (**a**) and K14-rtTA/TC/Rosa^LSL-dnMAML1^ (OS^LSL-dnMAML1^) (**b**) which had been fed with Dox from P0 to P16 and then changed to regular chow from P17 to P180. Eye photographs (**a**,**b**) were taken on P180. Noticed that severe ocular surface ulceration was observed in Noted that severe ocular surface ulceration was observed in OS^dnMAML1^ (compare **b** to **a**). Histological examinations on paraffin sections (**c**, **C**_**1**_, **d**, **D**_**1**_, **D**_**2**_) demonstrated that OS^dnMAML1^ (compare **d** to **c** ) led to persistent corneal edema, and neovascularization (**D**_**1**_) and conjunctival squamous hyperplasia (**D**_**2**_). Abbreviations: bv, blood vessel; cjs, conjunctival sac. (Courtesy of Development)

**Fig. 2:**
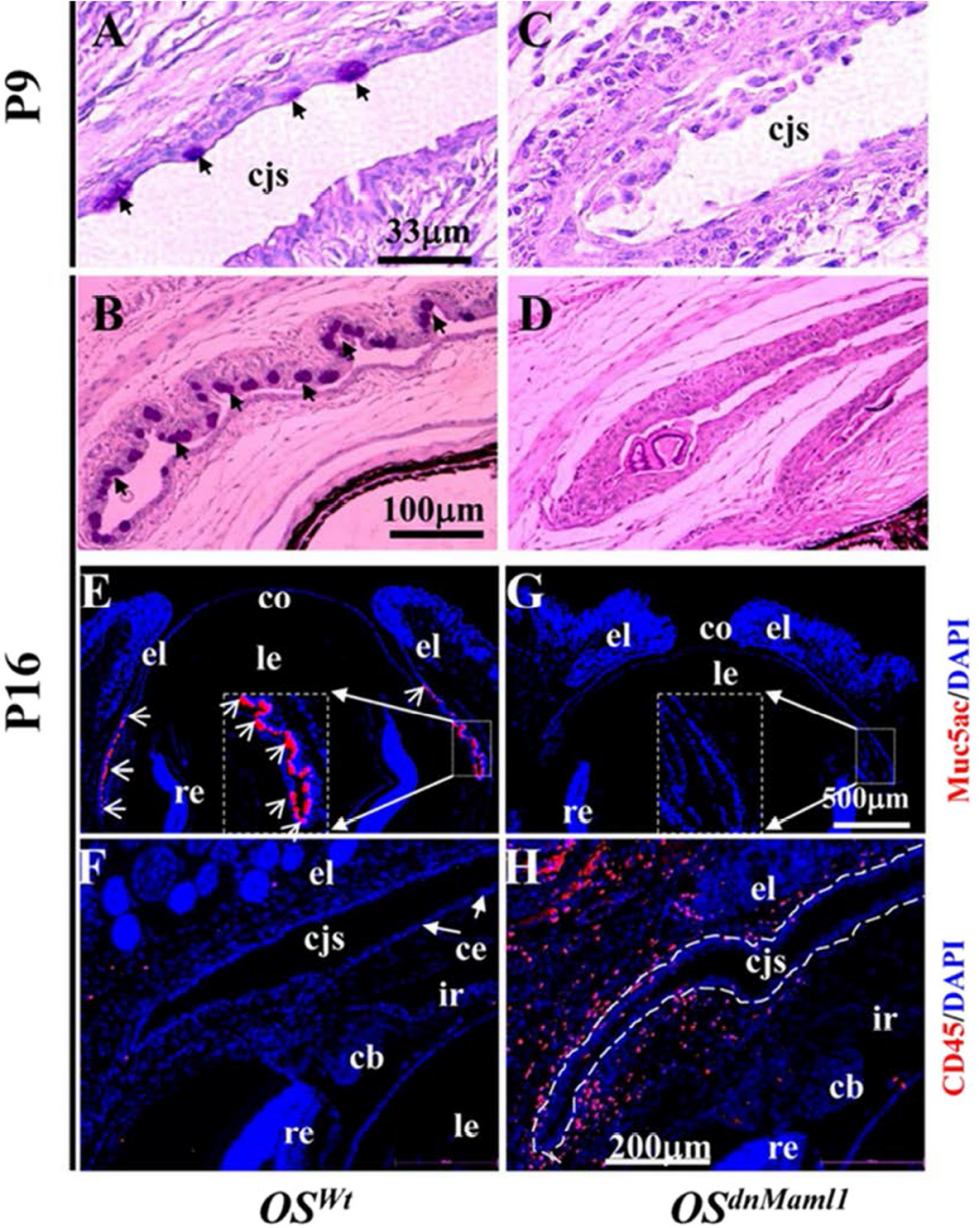
Expression of dnMaml1 inhibited conjunctival goblet cell differentiation and induced inflammation. **a**,**b** Periodic acid Schiff’s (PAS) and Hematoxylin staining showed that goblet cells (arrows) became evident at P9 (**a**) and formed clusters at P16 (**b**) during postnatal eye development in mice. **c**,**d** However, OS^dnMaml1^ revealed hyperplasia in conjunctival epithelium and failed to form goblet cells. **e**,**g** Conjunctival goblet cells were identified by positive signals (arrows) using anti-Muc 5a/c antibody. Most Muc5ac^+^ goblet cells clustered around forniceal and bulbar conjunctival region in P16 (**e**). By contrast, the expression of dnMaml1 led to total absence of Muc5ac^+^ goblet cells in the same region (**g**). **f**,**h** In addition, very few CD45^+^leukocytes were detected in the naïve ocular surface (**f**). By contrast, the expression of dnMaml1 led to abundant CD45^+^leukocyte infiltration into the subconjunctival space (**h**). cb, ciliary body; cjs, conjunctival sac; co, cornea; el, eyelid; ir, iris; le, lens; re, retina. (Courtesy of Development)

The dnMAML1 is a 62 amino acid peptide of the N-terminal basic domain (BD) of MAML1 that is capable of interacting with NICD, but it lacks the p300 transactivation domain (TAD) for histone acetylytransferase (HAT) binding capacity. Therefore, dnMAML1 is a pan-Notch inhibitor that interferes with the endogenous function of MAML proteins and inhibits transcriptional activation of all four Notch receptors [18]. The choice of K14rt/TC driver mouse allows genetic perturbation to be limited to stratified epithelia expressing K14, epidermal epithelium, hair follicle, ocular surface epithelia, but not in mesenchymal cells or simple epithelium in which MAML1 may have other functions than mediating Notch signaling. In addition, genetic perturbation can only take place when the experimental mice are induced by Dox administration. Thus this model provides advantage of spatial-temporal regulation of Notch signaling in ocular surface tissue morphogenesis. Indeed, upon Dox treatment, excess dnMAML1 was induced to compete with endogenous MAMLs binding to NICD/RBP-Jκ complex in K14-positive stratified epithelia including ocular surface. We found that expression of dnMAML1 in ocular surface (OS^dnMAML1^) in Dox-treated K14-rt/TC/Rosa^LSL-dnMAML1^ triple transgenic mice manifested conjunctival epithelial hyperplasia, aberrant desquamation, and impairment of goblet cell differentiation (Fig. 1). It should be noticed that, in this mouse model, we did not detect inflammation in OS^dnMAML1^ mice at P9, but 3 – 4 days after eyelid open at P16, profound CD45+ leukocyte infiltration was found in the sub-conjunctival stroma as compared to that in OS^Wt^ littermates (Fig. 2). These data indicated that the impairment of goblet cell formation was prior to sub-conjunctival inflammation in OS^dnMAML1^ mice. These results indicated that intrinsic canonical Notch signaling pathway played an important role in the goblet cell differentiation during ocular surface morphogenesis. Moreover, we found that OS^dnMAML1^ resulted in a dramatic down-regulation of Klf-4 and Klf-5 expression in bulbar/forniceal conjunctiva [18]. Interestingly, transfection of mN1-ICD cDNA enhanced mKlf4 promoter activity by 2 to 4.5 fold; likewise, transfection of Klf4 cDNA boosted mMuc 5/ac promoter activity by greater than one thousand fold in HEK293 cells. These data were consistent with the in vivo observations in which OS^dnMAML1^ resulted in complete loss of goblet cells and dramatic downregulation of protein and mRNA levels of Klf4 and Klf5 in the conjunctival epithelium. Collectively, our data implied that fine-tuning of Klf4 by Notch activity can greatly augment Muc 5/ac gene expression and thus goblet cell differentiation.

### Summary

**Fig. 3:**
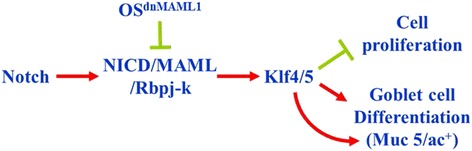
A proposed model depicts that canonical Notch signaling augments the expression of Klf4/5, resulting in conjunctival goblet cell differentiation by down-regulating proliferation and up-regulating transcription of Muc 5/ac in conjunctival epithelium. However, OS^dnMAML1^ blocks canonical Notch pathway, down-regulates Klf4/5, impairs Muc 5/ac synthesis and abrogates goblet cell differentiation (Courtesy of Development)

Identification of Jagged1 → Notch1 → N1-ICD/Rbp-jκ/MAML1 → Klf4/5 → Muc 5/ac axis (Fig. 3) for conjunctival goblet cell differentiation is a novel observation. Li et al. [19] recently reported that Ras-MAPK-ERK signaling pathway used by growth factor (such as EGF) can stimulate goblet cell proliferation *ex vivo*. Heuberger et al. [20] showed that Shp2/MAPK signaling controls goblet/paneth cell fate decisions in the intestine. We have also shown that the goblet cell density was significantly reduced in K14-derived conjunctival epithelium of the Shp2-conditional knockout (*Shp2*^*cko*^) mice [21]. Hence, perhaps Shp2-mediated activation of ERK, Src, and PI-3K/AKT signaling pathways also contribute to the conjunctival goblet cell proliferation, differentiation, and Muc 5/ac production. It is interest to investigate if Shp2-Ras-ERK, Shp2-Src, and/or Shp2-PI-3K/AKT can interact with Notch signaling. Further studies should reveal a signal network concerning conjunctival goblet cell growth and differentiation in vivo.

### Ethics approval

All the mice were bred at the Animal Facility of the University of Cincinnati Medical Center. Experimental procedures for handling the mice were approved by the Institutional Animal Care and Use Committee, University of Cincinnati/College of Medicine.
